# Online and SMS gateway MPS (Making Pregnancy Safer) as a form of institutional coalition to lower maternal mortality (AKI) and infant mortality (AKB) in Kulon Progo Regency

**DOI:** 10.1186/1471-2458-14-S1-P18

**Published:** 2014-01-29

**Authors:** Emmy Nirmalasari, Albertus Sunuwata, RN Indra Komala

**Affiliations:** 1 Universitas Gadjah Mada, Yogyakarta 55281, Indonesia

## Background

Internet as a source of information has developed, especially in the medical field. In developed countries, over 50 million people access the internet to discover their heath information. In Nigeria, teenagers use the internet as a source to find information on their reproductive health. Indonesia as a developing country also competes in developing online information system. With the development of online system involving internet connectivity to help referral system, an idea on online and SMS gateway Making Pregnancy Safer (MPS) to support easier, faster and more accurate information system was proposed by the Department of Health of Kulon Progo Yogyakarta as an effort to lower the maternal and infant mortality in the area. Both systems are forms of coalition between Department of Health and other health agencies such as public health centres, hospitals, and private health cares. The purpose of this study was to show that the online and SMS Gateway work system as an innovation of data collection support system in referral system might solve obstetric emergencies in expectant mothers and high-risk expectant mothers to lower maternal and infant mortality in Kulon Progo Regency in Yogyakarta.

## Materials and methods

This was a case study using descriptive analysis. The researchers performed observation in the field on expectant and high-risk mothers sample in Kulon Progo Yogyakarta.

## Results

Since its implementation in 2010, there were 6 less cases of maternal mortality, and in 2012 there were 3 less cases. For infant mortality, there were 44 less cases in 2010 and in 2012 there were 4 less cases. The cooperative process in the two systems showed in the reports from cadres to private midwives (BPS), from BPS to Department of Health (Dinkes), and Department of Health to Hospitals.

## Conclusions

With online and SMS Gateway MPS, Kulon Progo Regency experienced slow decrease in maternal mortality and infant mortality, but it will reach international MDG target in 2015.

